# The study of brain functional connectivity in Parkinson’s disease

**DOI:** 10.1186/s40035-016-0066-0

**Published:** 2016-10-28

**Authors:** Lin-lin Gao, Tao Wu

**Affiliations:** 1 Department of Neurobiology, Key Laboratory on Neurodegenerative Disorders of Ministry of Education, Beijing Institute of Geriatrics, Xuanwu Hospital, Capital Medical University, Beijing, 100053 China; 2 Beijing Key Laboratory on Parkinson’s Disease, Parkinson Disease Center of Beijing Institute for Brain Disorders, Beijing, China

**Keywords:** Parkinson’s disease, Functional connectivity, fMRI

## Abstract

Parkinson’s disease (PD) is a neurodegenerative disorder primarily affecting the aging population. The neurophysiological mechanisms underlying parkinsonian symptoms remain unclear. PD affects extensive neural networks and a more thorough understanding of network disruption will help bridge the gap between known pathological changes and observed clinical presentations in PD. Development of neuroimaging techniques, especially functional magnetic resonance imaging, allows for detection of the functional connectivity of neural networks in patients with PD. This review aims to provide an overview of current research involving functional network disruption in PD relating to motor and non-motor symptoms. Investigations into functional network connectivity will further our understanding of the mechanisms underlying the effectiveness of clinical interventions, such as levodopa and deep brain stimulation treatment. In addition, identification of PD-specific neural network patterns has the potential to aid in the development of a definitive diagnosis of PD.

## Background

Parkinson’s disease (PD) is the second most common neurodegenerative disorder in the aging population. PD is characterized by progressive deterioration of motor function, such as bradykinesia, rigidity, resting tremor, gait disturbance, and postural instability. Patients with PD also experience non-motor symptoms such as cognitive deficits, anxiety, apathy, hallucination, and depression. The pathological hallmark of PD is progressive decrease in dopamine concentrations and neuronal cell loss within the substantia nigra and other brain structures combined with the appearance of intracytoplasmic inclusions composed of α-synuclein aggregates known as Lewy bodies [1]. However, the precise mechanism by which the pathological changes in the brain result in the described clinical symptoms is unknown. It is well known that PD affects a large scale of neural networks. For example, dysfunction of cortico-basal ganglia-thalamo-cortical pathway is well known to be critical for the development of parkinsonian symptoms [2]. Therefore, further examination of neuronal network integrity may provide more valuable information for understanding the pathophysiological changes of PD than investigations of local brain activity, and may be helpful to bridge the gap between pathological changes and clinical presentations in PD.

The development of techniques such as functional magnetic resonance imaging (fMRI), electroencephalography (EEG), magnetoencephalography MEG), and transcranial magnetic stimulation (TMS) has greatly enhanced the ability to evaluate functional network integrity in vivo [3, 4]. In recent years, extensive studies have investigated PD-related disruption of functional networks, and have provided useful information regarding neurophysiological mechanisms underlying parkinsonian symptoms. In addition, these studies have served to identify mechanisms of anti-parkinsonian interventions, and suggest that brain networks have the potential to be developed as a biomarker for the diagnosis of PD. The aim of this review is to provide a comprehensive overview of the application of functional network connectivity in investigating neural mechanisms underlying parkinsonian symptoms and interventions, and as a potential biomarker in patients with PD. The reviewed publications were selected by the authors on the basis of relevance to the topic. The functional connectivity studies included in the current study is summarized in Table 1.

**Table 1:** Summary of the characteristics of the reviewed studies

Paper	Number of patients	Task	Type of connectivity	Main findings
Wu T et al., 2011 [12]	18 PD patients	A self-initiated right hand tapping task	Effective connectivity psychophysiological interaction (PPI)	The striatum-cortical connections were weakened, while the cortico-cerebellar connections were strengthened in PD
	18 controls			
Rowe J et al., 2002 [13]	12 PD patients	An overlearned motor sequence task, with and without attention	Effective connectivity • Structural equation modeling (SEM)	Attention to action did not increase the connectivity between the prefrontal cortex, lateral premotor cortex and SMA in PD
	12 controls			
Wu T et al., 2010 [14]	12 patients	two sequences of right hand finger tapping	Effective connectivity psychophysiological interaction (PPI)	The pre-SMA, cerebellum, and cingulate motor area had increased effective connectivity with brain networks in PD
	12 age-and sex-matched healthy subjects			
Rowe JB et al., 2010 [15]	16 PD patients	A visually paced finger-tapping task	Effective connectivity Dynamic causal modelling (DCM)	The coupling between the prefrontal cortex and the pre-SMA was enhanced in PD
	42 controls			
Wu T et al., 2016 [27]	36 PD patients	Handwriting	Functional connectivity	Dysfunction of basal ganglia motor circuit in both consistent and progressive. Progressive micrographia was also associated with disconnections between the pre-SMA, rostral cingulated motor area and cerebellum
	18 controls			
Wu T et al., 2015 [22, 29]	22 PD patients	Visuomotor association task	Effective connectivity Granger causality analysis (GCA)	The connectivity from the putamen to the motor cortex was decreased in PD
	22 controls			
Ma H et al., 2015 [30]	50 PD patients	Resting state	Functional connectivity	The bilateral dentate nucleus had higher connectivity with the bilateral cerebellar anterior lobe, and lower connectivity with the bilateral prefrontal cortex in tremor-dominant PD
	29 age-matched health controls			
Tessitore A et al., 2012 [32]	29 PD patients	Resting state	Functional connectivity	Reduced connectivity within both executive-attention and visual networks
	15 controls			
Liu H et al., 2013 [34]	9 PD patients	Resting state	Functional connectivity	Decreased connectivity of the dentate nucleus with the bilateral cerebellar posterior lobe in tremor-dominant PD
	9 controls			
Hu X et al., 2015 [35, 43]	21 tremor-dominant (TD)-PD	Resting state	Voxel-mirrored homotopic connectivity (VMHC)	TD-PD exhibited significantly lower VMHC values in the posterior lobe of the cerebellum. AR-PD exhibited lower VMHC values in the precentral gyrus.
	29 akinetic-rigid (AR)-PD patients			
	26 controls			
Seibert TM et al., 2012 [36]	19 cognitively unimpaired controls, 19 cognitively unimpaired PD patients,	Resting state	Functional connectivity	Decreased striato-prefrontal connectivity in patients with dementia
	18 patients with dementia			
Gorges M et al., 2015 [37]	14 cognitively unimpaired PD patients, 17 cognitively impaired PD patients	Resting state	Functional connectivity	Decreased default mode network connectivity in cognitively impaired PD patients.
	22 controls			
Disbrow EA et al., 2014 [38]	14 non-demented PD patients, 20 controls.	Resting state	Functional connectivity	Decreased default mode network connectivity in PD
Manza P et al., 2016 [41]	62 early-stage PD patients	Resting state	Functional connectivity	Motor deficit was associated with weaker coupling between anterior putamen and midbrain, cognitive impairment was associated with stronger coupling between the dorsal caudate and the rostral anterior cingulate cortex
Luo C et al., 2014 [42]	29 PD patients with depression, 30 PD patients without depression, 30 controls	Resting state	Functional connectivity	Reduced connectivity in the prefrontal-limbic network in the depression group
Hu X et al., 2015 [35, 43]	20 depressed PD patients, 40 non-depressed PD patients, 43 controls	Resting state	Functional connectivity	Stronger connectivity between the left median cingulate cortex and default mode network in the depressed PD
Sunwoo MK et al., 2015 [46]	110 PD patients subdivided into three groups based on olfactory performance	Resting state	Functional connectivity	Enhancement of striatocortical connectivity in the bilateral occipital areas and right frontal areas in patients with olfactory impairment
Baggio HC et al., 2015 [47]	62 PD patients, 31 controls	Resting state	Functional connectivity	Reduced connectivity in left-sided circuits, predominantly involving limbic, striatal and frontal territories in apathetic PD patients
Yao N et al., 2015 [48]	12 PD patients without hallucinations, 12 PD patients with visual hallucinations, 14 controls	Resting state	Functional connectivity	Increased occipital-corticostriatal connectivity in PD patients with visual hallucinations
Kwak Y et al., 2010 [50]	24 mild to moderate stage PD patients, 24 controls	Resting state	Functional connectivity	Increased cortico-striatal connectivity in PD patients
Agosta F et al., 2014 [51]	69 PD patients, 25 drug-naïve, 44 dopamine treated, 27 controls	Resting state	Functional connectivity	Decreased striato-thalamic connectivity, increased striato-temporal, and thalamo-cortical connections in dopaminergic treated PD
Bell PT et al., 2015 [52]	39 PD patients, controls	Resting state	Functional connectivity	Decoupling between the striatum and thalamic and sensorimotor networks in PD
Szewczyk-Krolikowski K et al., 2014 [53]	19 PD patients, 19 controls	Resting state	Functional connectivity	Reduced basal ganglia network connectivity in PD
Herz DM et al., 2015 [54]	26 PD patients	Visually cued movement	Effective connectivity Dynamic causal modelling (DCM)	Increase in the putamen and primary motor cortex connectivity after levodopa intake during movement suppression in patients who later developed levodopa-induced dyskinesias
Herz DM et al., 2016 [55]	12 PD patients with dyskinesias, 12 patients without dyskinesias	Resting state	Functional connectivity	Increased connectivity between the primary sensorimotor cortex and putamen after levodopa intake in patients with dyskinesias
Kahan J et al., 2014 [57]	12 PD patients	Resting state	Effective connectivity Dynamic causal modelling (DCM)	The strength of effective subthalamic nucleus afferents and efferents were reducedm cortico-striatal, thalamo-cortical and direct pathways were strengthened by DBS
Schweder PM et al., 2010 [58]	1 PD patient	Resting state	Functional connectivity	Normalization of pathological pedunculopontine nucleus (PPN) connectivity after PPN-DBS
Long D et al., 2012 [59]	19 early PD patients, 27 controls	Resting state	RFCS (regional functional connectivity strength)	The PD patients showed significant RFCS increases in the left parahippocampal gyrus, left angular gyrus and right middle temporal gyrus

## Techniques to assess network integrity

EEG, fMRI, and MEG are the most widely used techniques that enable researchers to assess large-scale neural networks at different spatial and temporal resolutions. With the advantages of being noninvasive and having high spatial resolution, fMRI is now the most used method to investigate functional integrity of networks in PD. In broad definition, fMRI includes all magnetic resonance imaging (MRI) methods that detect neural functional changes, such as blood oxygen level-dependent (BOLD) contrast imaging, perfusion, or diffusion. However, fMRI typically refers to BOLD fMRI, which detects changes in oxygen saturation levels of the blood [5]. In this review, the applications of BOLD fMRI on network integrity in PD will be discussed. The methods used to explore network integration involve the analysis of functional or effective connectivity [6–10]. Functional connectivity is defined as a temporal correlation between spatially remote neurophysiological events, whereas effective connectivity is defined as the influence that one neuronal system exerts over another [11]. Findings from both methods will be presented in this review.

## Motor symptoms-related network changes

### Bradykinesia

Bradykinesia is an important feature contributing to motor difficulties in PD. In this review, we use bradykinesia to describe bradykinesia (slowness of movement), hypokinesia (smallness of movement), and akinesia (lack of movement). Although extensive research has been conducted in this area, the pathophysiological mechanisms underlying bradykinesia remain unclear. Several neuroimaging studies have investigated network connectivity during performance of various motor tasks in patients with PD. During the performance of self-initiated movement, the functional connectivity between the striatum and cortical motor areas, i.e., primary motor cortex (M1), premotor cortex (PMC), and supplementary motor area (SMA), is weakened in PD [12]. In addition, the connectivity between the prefrontal cortex, PMC and SMA is disrupted [12–15]. The SMA is critical in planning and decision of movements and plays a primary role in the preparation of self-initiated movements [16–18]. The SMA is one of the main receiving regions of the basal ganglia motor circuit [19]. The dysfunction of the SMA has been correlated with motor difficulty, and the administration of levodopa has been shown to relatively normalize the function of the SMA in patients with PD resulting in improved motor performance [20, 21]. Thus, the disconnection of the striato-SMA pathway due to the deficit of the nigrostriatal dopamine system is likely to be an important factor contributing to bradykinesia in PD.

Motor automaticity has been proposed as a possible mechanism underlying bradykinesia [22]. Automaticity is the ability to perform movements without attention directed toward the details of the movement [23]. In healthy people, the processing of motor automaticity is accompanied by the more efficiency of neural network and less significant of attentional network. The automated motor program is likely stored in the sensorimotor striatum (posterior putamen), and is resistant to interference [22, 24–26]. Most bradykinesia-related motor problems are associated with deterioration of motor automaticity, as PD patients tend to perform all daily behaviors slower or with smaller amplitude, e.g., akinesia, reduced arm swing, freezing of gait (FOG), and micrographia [27]. Motor automaticity dysfunction is already apparent in the early stages of PD [28, 29]. During automatic processing, the connectivity of striato-cortical motor pathways is decreased, the activity in the sensorimotor striatum is not enhanced, and the attentional networks remain active in PD compared to controls [22, 29, 30].

Based on these studies of neural networks, neural mechanisms for impaired motor automaticity in PD includes less efficient neural coding of movement, failure to shift automated motor skills to the sensorimotor striatum, instability of the automatic mode within the striatum, and use of attentional control efforts to execute movements usually performed automatically in healthy people [22]. As a consequence, PD patients lose previously acquired automatic skills and have difficulty in acquiring new automatic skills or restoring lost motor skills, which results in bradykinesia.

### Tremor

As tremor may disturb fMRI signals, tremor-related network connectivity has been much less investigated compared to bradykineisa. In an elegant study, Helmich and colleagues described the use of electromyography to monitor tremor during fMRI scanning, and measured functional connectivity between basal ganglia nuclei and the cerebellothalamic circuit [31]. The authors reported that the basal ganglia nuclei were transiently activated at the onset of tremor, while activity in the cerebellothalamic circuit correlated with tremor amplitude. The internal globus pallidus and putamen had increased functional connectivity with the cerebellothalamic circuit. These findings suggest that parkinsonian tremor may result from a pathological interaction between the basal ganglia and the cerebellothalamic circuit, which is supported by the following studies [30].

Functional connectivity experiments have also been used to explore the underlying mechanisms for several other parkinsonian motor symptoms. For example, Tessitore and colleagues reported that PD patients with FoG had impaired functional connectivity within the frontoparietal networks sub-serving attentional functions [32]. Functional neuroimaging studies suggest that the disturbances in frontal cortical regions, the basal ganglia, and the midbrain locomotor region are possibly the origins of FoG [33]. Network connectivity also can be used to identify the neural characters in different subtypes of PD [2, 34, 35].

## Non-motor symptoms

In addition to symptoms related to motor function, most PD patients present with some non-motor symptoms such as cognitive, emotional, or olfactory impairments. In recent years, more focus has been applied to characterizing the neural network of these non-motor symptoms. Cognitive deficits are common in PD patients. PD with dementia is associated with selective disruption of corticostriatal connectivity [36]. Moreover, it has been shown that the connectivity of the so called “default mode network” (DMN) is disrupted in PD patients with cognitive deficits [37, 38]. The DMN is a network showing consistent task-related deactivations, and includes the medial prefrontal cortex, anterior cingulate cortex, posterior cingulate cortex, precuneus, and inferior parietal lobe [39, 40]. The DMN is thought to facilitate cognitive performance by allocating neural resources to critical brain regions. The disruption of the DMN was associated with the progress of cognitive decline [37], while the decline in cognitive function, particularly in the memory and visuospatial domains, was associated with stronger coupling between the dorsal caudate and the rostral anterior cingulate cortex [41]. These findings suggest that malfunctioning of the DMN may contribute to the executive function deficits in PD.

Depression is the most frequent psychiatric disorder reported in patients with PD. Abnormal prefrontal-limbic network connectivity has been demonstrated in depressed PD patients [31, 42]. PD patients with depression are associated with disrupted functional connectivity between the median cingulate cortex and precuneus, prefrontal cortex, and cerebellum [43]. The cingulate cortex plays key roles in integrating multimodal information that is important for emotional, sensorimotor, and cognitive functions [44]. The median cingulate cortex also appears to be involved in many emotion-related cognitive processes such as meditation, self-related rumination, aversive conditioning, and the anticipation and perception of pain [45]. The impaired median cingulate cortex-related networks may play a role in depression experienced by patients with PD.

Network connectivity in some other non-motor symptoms, such as olfactory impairment, apathy, and hallucination has also been investigated. PD patients with olfactory impairment had decreased connectivity between the posterior cingulate cortex and bilateral primary sensory areas, right frontal areas, and right parietal areas, and had an enhancement of striatocortical connectivity compared to PD patients with normal olfaction [46]. Apathetic PD patients showed reduced functional connectivity mainly involving limbic striatal and frontal territories. In addition, the limbic division of the left striatum showed reduced connectivity with the ipsilateral frontal cortex and with the rest of the left striatum [47]. In PD patients with visual hallucinations, occipital-cortico-striatal connectivity was significantly higher than in patients without hallucinations [48]. Hallucinations have been associated with functional abnormalities in primary visual cortex and visual associative cortices [49].

## Intervention-related network changes

Functional connectivity can be also used to investigate neural mechanisms underlying anti-parkinsonian interventions. Levodopa treatment has been reported to normalize the function of the basal ganglia motor pathways (e.g., by enhancing neural activity in the SMA and striatum) and restore striato-cortical motor pathway connectivity [4, 50–53] in a manner associated with improvements in motor function.

Although levodopa remains the most effective medication for the management of PD symptoms, many PD patients develop daily fluctuations in mobility and involuntary movements known as levodopa-induced dyskinesias (LID). The neural correlates in the genesis of LID remain poorly understood. A recent study found an increase in connectivity between the putamen and M1 after levodopa intake in patients developed LID [54]. This excessive striato-cortical connectivity in response to levodopa may play a role in the pathophysiology of LID [54, 55]. Another study showed that the connectivity of inferior frontal cortex was decreased with M1 and increased with the putamen in patients with LID [56]. This finding suggests that the neural network centered on the inferior frontal cortex may also involve in the pathophysiological mechanisms underlying LID.

Deep brain stimulation (DBS) is another effective therapy for PD, but the neural mechanism underlying therapeutic effects of DBS remain unclear. It has been shown that DBS on the subthalamic nucleus (STN) can modulate the connectivity of striato-thalamo-cortical and STN-cortical pathways in association with symptom improvements [57]. The pedunculopontine nucleus (PPN) is a target in treating primarily gait and posture symptoms. PPN-DBS has been reported to normalize pathological PPN connectivity [58].

## Diagnosis

The diagnosis of PD is based mainly on clinical assessments. Some studies have combined fMRI and various pattern analysis methods to try to establish an imaging methodology for PD diagnosis [53, 59–61]. In a recent study, the authors of this review have identified a PD-related spatial covariance pattern that was characterized by decreased activity in the striatum, supplementary motor area, middle frontal gyrus, and occipital cortex, and also by increased activity in the thalamus, cerebellum, precuneus, superior parietal lobule, and temporal cortex. This pattern had a high accuracy (90 %) to discriminate PD patients from healthy controls [59]. These studies have proven that network connectivity approach can identify characteristic PD-specific neural changes, and has the potential of network pattern as a biomarker for PD diagnosis. However, functional connectivity cannot directly reveal pathological changes in PD, therefore, whether this method can be applied in clinical practice needs further investigation.

## Conclusions

### Future directions

Studies on functional connectivity have provided important information on PD-related functional and pathophysiological changes. At present, functional connectivity is primarily used to further understand how pathological changes lead to parkinsonian symptoms, and is far from being a method in routine clinical investigations. As shown in the Table 1, the analytic methods of "functional connectivity" studies vary a lot from study to study. Few studies have used the same "functional connectivity" procedure. Therefore, it is hard to perform meta-analysis on these studies. In contrast, voxel-level analysis, like most PET studies and some resting-state fMRI studies focusing the local activity support coordinate-based meta-analysis and, hence, are more helpful to clinical studies. Additional research should focus on increased efforts to develop neural network pattern as a neuroimaging biomarker for early diagnosis of PD; this might well require further technical and methodological improvements. These developments will improve early diagnosis, better evaluate disease progression, differentiate PD from other parkinsonisms on an individual basis, and may guide novel targets for future therapies.
